# A Predictive Coding Perspective on Mismatch Negativity Impairment in Schizophrenia

**DOI:** 10.3389/fpsyt.2020.00660

**Published:** 2020-07-08

**Authors:** Kenji Kirihara, Mariko Tada, Daisuke Koshiyama, Mao Fujioka, Kaori Usui, Tsuyoshi Araki, Kiyoto Kasai

**Affiliations:** ^1^Department of Neuropsychiatry, Graduate School of Medicine, The University of Tokyo, Tokyo, Japan; ^2^International Research Center for Neurointelligence (WPI-IRCN), UTIAS, The University of Tokyo, Tokyo, Japan; ^3^Department of Psychiatry, University of California, San Diego, San Diego, CA, United States

**Keywords:** mismatch negativity, schizophrenia, electroencephalography, predictive coding, computational modeling

## Abstract

Mismatch negativity (MMN) is a widely used biological marker for schizophrenia research. Previous studies reported that MMN amplitude was reduced in schizophrenia and that reduced MMN amplitude was associated with cognitive impairments and poor functional outcome in schizophrenia. However, the neurobiological mechanisms underlying the reduced MMN amplitude remain unclear. Recent studies suggest that reduced MMN amplitude may reflect altered predictive coding in schizophrenia. In this paper, we reviewed MMN studies that used new paradigms and computational modeling to investigate altered predictive coding in schizophrenia. Studies using the roving oddball paradigm and modified oddball paradigm revealed that the effects of conditional probability were impaired in schizophrenia. Studies using omission paradigms and many-standards paradigms revealed that prediction error, but not adaptation, was impaired in schizophrenia. A study using a local-global paradigm revealed that hierarchical structures were impaired at both local and global levels in schizophrenia. Furthermore, studies using dynamic causal modeling revealed that neural networks with hierarchical structures were impaired in schizophrenia. These findings indicate that altered predictive coding underlies the reduced MMN amplitude in schizophrenia. However, there are several unsolved questions about optimal procedures, association among paradigms, and heterogeneity of schizophrenia. Future studies using several paradigms and computational modeling may solve these questions, and may lead to clarifying the pathophysiology of schizophrenia and to the development of individualized treatments for schizophrenia.

## Introduction

Schizophrenia is a psychiatric disorder characterized by positive symptoms such as delusion and hallucination, and negative symptoms such as impaired motivation and social withdrawal, and cognitive impairment. The onset of schizophrenia is usually in adolescence and early adulthood, and many patients experience long-term impairments in social and occupational functions (1, 2).

Mismatch negativity (MMN) is a widely used biological marker in schizophrenia research. MMN amplitude is reduced in schizophrenia, first-episode schizophrenia, and ultra-high risk (3). MMN latency is also altered in schizophrenia and ultra-high risk (4). Small MMN amplitude found in individuals at ultra-high-risk predicts the future onset of psychosis (5) and future remission (6). Reduced MMN amplitude is associated with cognitive impairments and poor functional outcomes (7), but not associated with genetic vulnerability (8). Therefore, the investigation of mechanisms underlying reduced MMN in schizophrenia may lead to an understanding of the pathophysiology of schizophrenia and the development of more effective treatments.

**Figure 1:**
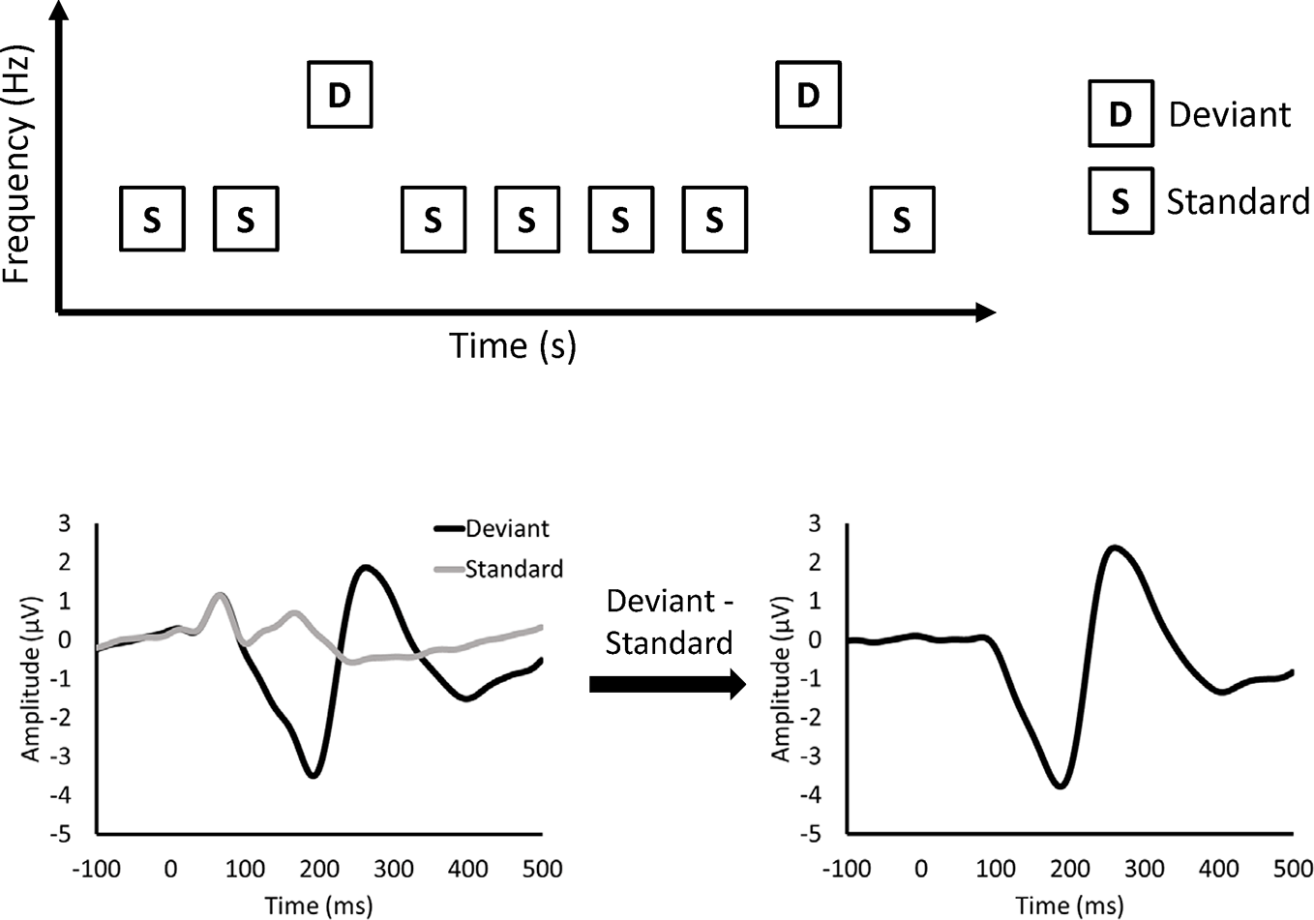
Classical oddball paradigm and mismatch negativity.

MMN is measured by electroencephalography (EEG) or magnetoencephalography (MEG) recorded during a passive auditory oddball paradigm (**Figure 1**). In the oddball paradigm, deviant and standard stimuli are randomly presented. Deviant stimuli are rare (e.g., 10%), while standard stimuli are frequently presented (e.g., 90%). In the figures of this article, deviant and standard stimuli differ in frequency. However, other features, such as duration, can be used to differentiate between deviant and standard stimuli. MMN is obtained as a negative deflection of the difference waveform between the event-related potentials (ERPs) elicited by deviant and standard stimuli. MMN was originally thought to reflect sensory memory (9). In the sensory memory hypothesis, the memory of auditory stimuli is formed in the auditory cortex, and MMN is elicited by comparison between standard and deviant stimuli. Another explanation for the MMN is the adaptation hypothesis (10). In the adaptation hypothesis, as neuronal responses to standard stimuli decline after repetitive presentation of standard stimuli (11), MMN is thought to represent the difference between intact neural responses to deviant stimuli and attenuated neural responses to standard stimuli. Recently, MMN was thought to reflect the prediction error based on predictive coding theory (12).

## Predictive Coding Account for MMN in Schizophrenia

In predictive coding theory, the brain generates a model to infer the causes of sensory inputs, predicts sensory inputs based on the model, calculates the differences between prediction and sensory inputs, and updates the model based on prediction error (13). Alterations in these processes generate a maladaptive model and make a false inference that leads to hallucination and delusion. Several studies reported that patients with auditory hallucinations put more weight on predictions than on sensory evidence when compared with healthy individuals (14, 15). Relying more on prediction rather than on sensory evidence may create false beliefs that lead to psychosis (16). In fact, enhanced prediction correlated with positive symptoms in patients with schizophrenia (17). However, other studies reported that patients with schizophrenia put little weight on prediction and much weight on sensory evidence (18, 19). Too much weight on sensory evidence may contribute to aberrant salience, which also leads to psychosis (20). Because many studies have reported altered predictive coding in schizophrenia (21), it is important to investigate neurobiological mechanisms underlying altered predictive coding in schizophrenia.

MMN is a candidate biomarker for investigating neurobiological mechanisms underlying altered predictive coding in schizophrenia. Several studies have shown that the predictive coding theory not only explains MMN (22) but also provides a more plausible model than the sensory memory hypothesis or the adaptation hypothesis (23). Because MMN can serve as a translational biomarker (24), animal studies measuring MMN can be used to investigate neurobiological mechanisms underlying altered predictive coding in schizophrenia.

Based on these findings, recent studies have explained reduced MMN as altered predictive coding in schizophrenia (12, 24). Furthermore, several studies have developed new paradigms to investigate whether reduced MMN amplitudes reflect altered predictive coding in schizophrenia (25–27). In this paper, we reviewed MMN studies that used new paradigms to investigate altered predictive coding in schizophrenia.

## Paradigms to Investigate the Predictive Coding Account of MMN in Schizophrenia

### Mismatch Negativity and Probability

MMN is considered to reflect the prediction error in predictive coding theory because MMN is elicited by deviant stimuli that are presented with low probability. As the brain usually predicts stimuli with high probability, stimuli with low probability do not match prediction and cause prediction errors. In the classical oddball paradigm, stimuli are randomly presented. If the probability of the next stimuli depends on previously presented stimuli, previously presented stimuli form predictions about what stimuli come next. Therefore, paradigms that have different conditional probabilities can manipulate prediction and prediction errors. Such paradigms are appropriate for investigating the association between prediction error and MMN. Several researchers have used paradigms with different conditional probabilities to investigate the effects of conditional probability on MMN.

#### Roving Oddball Paradigm

**Figure 2:**
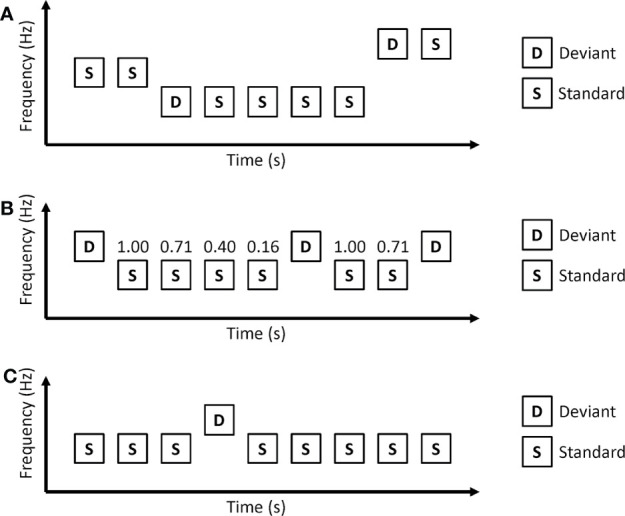
Oddball paradigms in which conditional probability of stimuli depend on the length of repetitive standard stimuli. **(A)** Roving oddball paradigm. **(B)** Modified oddball paradigm. The numbers above standard stimuli represent conditional probability. The conditional probability that standard stimuli come after a deviant stimulus is 1.00, the conditional probability that 2 consecutive standard stimuli come after a deviant stimulus is 0.71, and so on. **(C)** Predictable oddball paradigms. In the predictable condition, auditory stimuli are repetitions of a series of “SSSDSSSS.”.

In the roving oddball paradigm, auditory stimuli are presented as trains (**Figure 2A**). Each train consists of repetitions of the same auditory stimuli. Usually, the first tone of a train is used as a deviant, and the last tone of the train is used as a standard. As the roving oddball paradigm uses the same tones as deviant and standard stimuli, tone differences between deviant and standard stimuli do not affect MMN in this paradigm. Another important point of the roving oddball paradigm is that the prediction of deviant stimuli depends on the length of the repetitions. Long repetitions of standard stimuli increase the conditional probability of standard stimuli (conditional probability that a standard stimulus comes next after a standard stimulus is presented) and make a strong prediction that the next stimuli will be a standard stimulus. Therefore, deviant stimuli after long repetitions are thought to cause a strong prediction error.

Baldeweg and colleagues measured MMN using a modified version of the roving oddball paradigm (28). In their paradigm, the last tones of trains differ in duration compared to other stimuli and are used as deviant stimuli. They found that healthy controls showed larger MMN amplitudes under longer repetition conditions, while repetition showed no effect on MMN for patients with schizophrenia. Although the authors called repetition effects “memory trace effect” in this paper, they interpreted the repetition effects as evidence for predictive coding (29). They also reported that impairments in repetition effects were observed in patients with schizophrenia, but not with bipolar disorder or Alzheimer’s disease (30).

In the usual roving oddball paradigm, Schmidt and colleagues showed that S-ketamine reduced repetition effects (31). McCleery and colleagues investigated repetition effects on not only MMN but also ERPs for both standard and deviant stimuli (26, 32). They found reduced repetition effects in schizophrenia patients with recent auditory hallucinations when compared to schizophrenia patients without recent auditory hallucinations (32). However, they also found relatively intact repetition effects in all schizophrenia patients (26).

#### Modified Oddball Paradigm

The roving oddball paradigm uses the length of repetitions to generate different conditional probabilities of stimuli. However, this can be applied to the classical oddball paradigm. Ford and colleagues used a modified oddball paradigm that had different conditional probabilities according to the length of repetitions (33) (**Figure 2B**). In this paradigm, longer repetitions of standard stimuli correspond to a lower conditional probability that the next stimulus is a standard stimulus. Healthy control participants showed MMN to the 4th consecutive standard. The MMN to the 4th consecutive standard was reduced in patients with schizophrenia.

Horacek et al. investigated MMN in predictable and unpredictable conditions (**Figure 2C**) (34). In the predictable condition, deviant stimuli always came after 7 consecutive standard stimuli. The unpredictable condition is the same as a classical oddball paradigm in which deviant stimuli are randomly presented. Patients with schizophrenia showed a reduction in MMN amplitude in the unpredictable condition but no reduction in MMN amplitude in the predictable condition.

#### Summary of the Findings

All studies listed above showed significant effects of conditional probability on MMN in healthy individuals. These findings suggest that predictive coding can explain MMN. Many studies have shown reduced effects of conditional probability on MMN in patients with schizophrenia. These findings suggest that reduced MMN amplitude may reflect altered predictive coding in schizophrenia. While several studies have shown no difference in repetition effects between healthy individuals and patients with schizophrenia, others reported an association of repetition effects with cognitive impairments (28) and hallucinations (32). These results suggest that heterogeneity of schizophrenia may affect findings among studies. Patients with schizophrenia have an intact MMN in the predictable condition. Therefore, altered predictive coding in schizophrenia may become clear in uncertain environments.

### Prediction Error and Adaptation

All studies listed in 3.1. used repetitions to investigate the effects of conditional probability. However, repetitions of stimuli have been found to cause a reduced response to the stimuli (11). This is called adaptation which can explain repetition effects. Although adaptation and predictive coding are not mutually exclusive, the adaptation hypothesis is simple. If simple hypotheses such as adaptation can explain MMN sufficiently, more complex hypotheses such as predictive coding are unnecessary. Therefore, it is important to investigate MMN in paradigms that remove adaptation.

#### Omission Paradigm

**Figure 3:**
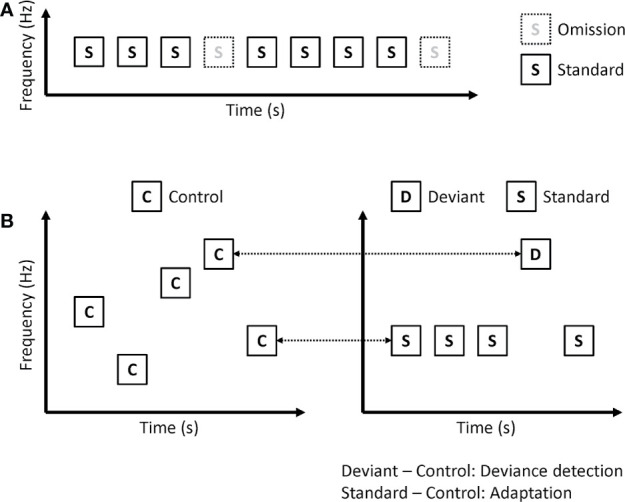
Paradigms that remove or disentangle adaptation effect on MMN. **(A)** Omission paradigm. **(B)** Many-standards paradigm (left). ERP to control is compared with the same tone in the classical oddball paradigm (right), and calculated as deviance detection and adaptation.

In omission paradigms, the same tones are repeatedly presented, but sometimes not presented (**Figure 3A**). Because deviants are no stimuli in omission paradigms, omission MMN is not affected by sensory processing including adaptation. Kreitschmann-Andermahr et al. showed reduced amplitude of omission MMN in schizophrenia (35). Rudolph and colleagues used more complex omission paradigms (36). In their paradigm, 6 consecutive tones constituted one train, and many trains were presented repeatedly. In the deviant trains, the 4th or 6th tones are omitted. They also found a reduced amplitude of omission MMN in early psychosis. However, Kirino et al. showed no significant reduction in omission MMN amplitude in schizophrenia (37).

#### Many-Standards Paradigm

Omission paradigms can remove adaptation effects on MMN. However, several studies have shown that patients with schizophrenia have impairments in neural adaptation (38). Therefore, investigating both prediction error and adaptation may be better than removing adaptation. Koshiyama and colleagues used a many-standards paradigm to disentangle MMN into adaptation and deviance detection (**Figure 3B**) (25). They recorded EEG during a classical oddball paradigm and a many-standards paradigm. In the many-standards paradigm, several different tones are randomly presented. In the classical oddball paradigm, one of the tones is the same as the deviant stimuli, while the other one is the same as the standard stimuli. Each tone in the many-standards paradigm is presented with equal probability, which is the same as the probability of deviant stimuli in the classical oddball paradigm. While standard stimuli are repetitively presented in the classical oddball paradigm, control stimuli are not repetitively presented in the many-standards paradigm. Therefore, the authors investigated adaptation by comparing ERP to standard stimuli in the classical oddball paradigm with ERP to control stimuli in the many-standards paradigm. Since deviant stimuli are detected as deviant in the classical oddball paradigm, but there is no deviance in the many-standards paradigm, they investigated deviance detection by comparing ERP to deviant stimuli in the classical oddball paradigm with ERP to control stimuli in the many-standards paradigm. They found that both adaptation and deviance detection affected MMN. Patients with schizophrenia showed impairments in deviance detection but not in adaptation.

#### Summary of Findings

Several studies have shown a reduction in the omission MMN amplitude in schizophrenia. As omission MMN reflects prediction error but not adaptation, these findings indicate that patients with schizophrenia have altered predictive processing. Another study that used the many-standards paradigm showed that both deviance detection and adaptation contributed to MMN while the reduction of MMN amplitude was mainly due to impairments of deviance detection in schizophrenia. Therefore, altered predictive coding rather than adaptation can explain the reduction of MMN amplitude in schizophrenia.

### Hierarchical Structure of Predictive Coding

In predictive coding theory, higher-level neurons send predictions to lower-level neurons. Lower-level neurons receive sensory inputs, compare prediction with sensory inputs, calculate prediction error, and send prediction error to higher-level neurons. Higher-level neurons modify prediction based on prediction error. Therefore, predictive coding is thought to depend on the hierarchical structure of neurons (39). Several paradigms and analysis methods have been developed to investigate the hierarchical structure of predictive coding.

#### Local-Global Paradigm

**Figure 4:**
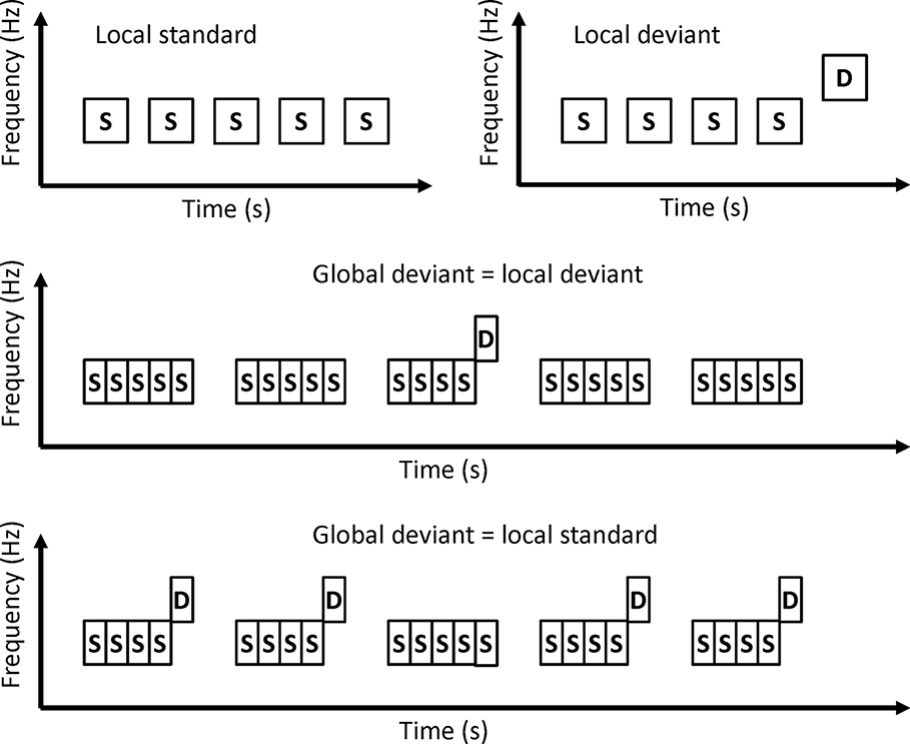
Local-global paradigm.

In local-global paradigms, a series of several tones are presented as trains (**Figure 4**). A train of identical tones is a local standard. A train in which the last tone is different from other tones is the local deviant. Local standards and local deviants are presented randomly. If a local standard is presented with a high probability, the local standard is the global standard and the local deviant is the global deviant. On the other hand, if a local deviant is presented with a high probability, the local deviant is the global standard, and the local standard is the global deviant.

Sauer et al. investigated MMN in a local-global paradigm (27). Patients with schizophrenia showed a reduction in MMN amplitude to both local deviants and global deviants. These findings suggest that patients with schizophrenia may have altered predictive coding at both local and global levels.

## Computational Modeling of Mismatch Negativity In Schizophrenia

### Dynamic Causal Modeling

**Figure 5:**
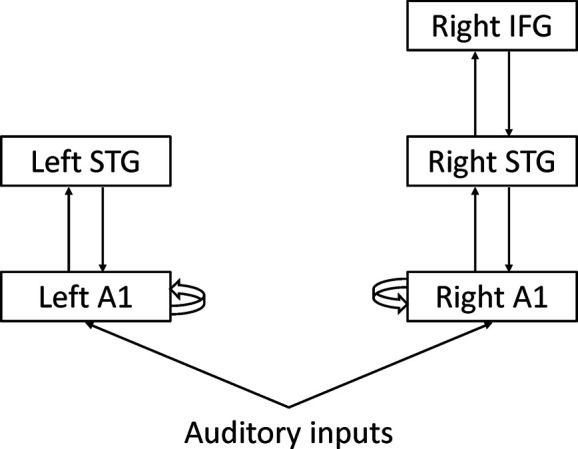
Dynamic causal modeling A1: Primary auditory cortex STG, Superior temporal gyrus IFG; Inferior frontal gyrus.

Dynamic causal modeling (DCM) is a network model initially developed for functional MRI data (40). The aim of DCM is to make inferences about coupling among brain regions and how that coupling is influenced by experimental factors. DCM calculates effective connectivity, defined as the influence that one brain region exerts over another brain region. DCM can be used for EEG data (41). Garrido and colleagues applied DCM to MMN and found that the hierarchical structure of cortical regions and connectivity among them could explain MMN (**Figure 5**) (42). From the perspective of predictive coding theory, forward connections from the low-level cortex to high-level cortex may convey prediction error, and backward connections from the high-level cortex to the low-level cortex may convey prediction. Intrinsic connections may reflect precision that determines the relative weight of prediction error to prediction. However, it remains unclear whether each connectivity corresponds to each process of predictive coding.

Dima and colleagues applied DCM to MEG data during the roving oddball paradigm (43). They found significant differences in the intrinsic connection of the right primary auditory cortex (A1) and backward connection from the right inferior frontal gyrus (IFG) to the right superior temporal gyrus (STG) between schizophrenia and healthy controls. Ranlund and colleagues applied DCM to EEG data during the classical oddball paradigm (44). They found significant differences in the intrinsic connections of the right IFG among patients with psychosis, unaffected relatives, and healthy individuals. Braeutigam and colleagues applied DCM to MEG data during the roving oddball paradigm (45). DCM selected different models among schizophrenia, bipolar disorder, and healthy controls.

All the DCM studies listed above supported complex models with hierarchical structures. These findings suggest that neural networks with hierarchical structures, but not single neurons or single neural circuits, underlie MMN. Patients with schizophrenia showed impairments of the neural network in most studies using DCM. However, neural network impairments in schizophrenia are inconsistent among studies.

## Discussion

Previous studies showed that conditional probability affected MMN, that neural adaptation could not explain MMN, and that neural networks with hierarchical structures underlay MMN. Patients with schizophrenia showed impairments in the effects of conditional probability, prediction error but not adaptation, both at local and global levels in the hierarchical structure, and neural networks in hierarchical structure. All these findings support the predictive coding account of MMN. However, there are several questions that remain unanswered.

One of these unsolved questions is the optimal procedure for each paradigm. For example, in the roving oddball paradigm, several studies used the last tone (different from standard stimuli) as deviant, but other studies used the first tone (same as standard stimuli) as deviant. Studies also reported that MMN in the classical oddball paradigm depends on stimulus, probability, and interstimulus interval (46, 47), so slight differences in procedures may lead to inconsistent findings among studies. For example, Koshiyama and colleagues investigated both duration-deviant MMN (dMMN) and frequency-deviant MMN (fMMN) to disentangle MMN into adaptation and deviance detection (25). Patients with schizophrenia showed significant impairments in deviance detection in dMMN but not in fMMN. Future studies like this one are necessary to investigate how differences in procedures affect MMN in various paradigms.

The second question is the association among various paradigms. Repetition effects in the roving oddball paradigm, omission MMN in omission paradigm, and deviance detection in many-standards paradigm are thought to reflect prediction error. However, it is unclear whether MMN in different paradigms reflects a common process of predictive coding or different processes of predictive coding. Because predictive coding assumes a complex and hierarchical structure, MMN in different paradigms may reflect different processes of predictive coding. All of the repetition effects, omission MMN, and deviance detection are thought to reflect prediction error. Adaptation affects repetition effects, but not omission MMN or deviance detection. Therefore, repetition effects may reflect the activity of lower-level neurons. Because there is no sensory input, omission MMN may reflect the activity of higher-level neurons. However, few studies have investigated the commonalities and differences among repetition effects, omission MMN, and deviance detection. Future studies are necessary to investigate the commonalities and differences of MMN in various paradigms.

The third question is the heterogeneity of schizophrenia (48). From the perspective of predictive coding theory, the neural mechanisms underlying MMN have a complex and hierarchical structure. Therefore, various kinds of impairments can cause altered predictive coding that leads to a reduction in MMN amplitude in schizophrenia. It is unlikely that all patients with schizophrenia have impairments in the same neural circuit. Rather, it is likely that impaired processes differ among patients with schizophrenia. Therefore, it is important not to detect single specific neural impairments in schizophrenia, but to identify individually different impairments. However, researchers can investigate only one aspect of predictive coding in one paradigm (e.g., repetition effect in the roving oddball paradigm).

Computational modeling may be useful for answering these questions. Computational modeling can extract information about various aspects of predictive coding from one paradigm (e.g., forward connections, backward connections, and intrinsic connections for dynamic causal modeling). Therefore, computational modeling may be useful for identifying individually different impairments in patients with schizophrenia. Computational modeling may also become a common framework for comparing different paradigms because computational modeling can be applied to various paradigms and can be compared statistically. In addition, computational modeling can be applied to the classic oddball paradigm, and many studies have already investigated the effects of the procedure. The most often used computational model of MMN in schizophrenia is dynamic causal modeling. Previous studies using DCM investigated which connections are impaired in schizophrenia. However, the heterogeneity of schizophrenia leads to inconsistent findings among studies. Previous studies reported impairments of intrinsic connections within the right A1, intrinsic connections within the right IFG, and backward connections from the right IFG to the right STG. For example, patients with impaired intrinsic connections of the right A1, patients with impaired intrinsic connections of the right IFG, and patients with impaired backward connections from the right IFG to the right STG may have different clinical characteristics associated with different neurobiological mechanisms. Therefore, DCM can serve as a biomarker for classifying subtypes of schizophrenia. Although previous studies have shown the utility of dynamic causal modeling, other computational models have not yet been applied to MMN in schizophrenia. Future studies using various computational models may be useful for identifying individual differences in predictive coding in schizophrenia.

MMN is considered a translatable biomarker because paradigms for the measurement of MMN can be applied to non-human animals (24). Several paradigms shown in this article have been already used for investigations of MMN in non-human animals (49–51). Therefore, the neurobiological mechanisms underlying each paradigm can be investigated in animal studies. MMN can also be measured with electrocorticography (ECoG), which has high spatial resolution. Investigation of ECoG with various paradigms is useful for identifying the precise location of neural circuits underlying MMN in various paradigms (52). In addition, MMN is used as a biomarker for clinical trials (53, 54). Identifying individually different impairments using several paradigms and computational modeling may lead to the development of individualized treatments.

In conclusion, previous studies using various paradigms and computational modeling showed that altered predictive coding underlies the reduction of MMN amplitude in schizophrenia. As neural mechanisms underlying predictive coding have a complex and hierarchical structure, impaired neural mechanisms may differ among patients with schizophrenia. Future studies using several paradigms and computational modeling may clarify the pathophysiology of schizophrenia and help the development of individualized treatments for schizophrenia.

## Author Contributions

KKi and KKa contributed to the conception of this review. KKi wrote the first draft of the manuscript. All authors contributed to the article and approved the submitted version.

## Funding

This work was supported, in part, by the Japan Society for the Promotion of Science (JSPS) KAKENHI (18K07588; KKi), the Brain Mapping by Integrated Neurotechnologies for Disease Studies (Brain/MINDS) from the Japan Agency for Medical Research and Development (AMED) (JP19dm0207069; KKa), the International Research Center for Neurointelligence (WPI-IRCN) at the University of Tokyo Institutes for Advanced Study (UTIAS) (MT, KKa), the University of Tokyo Center for Integrative Science of Human Behavior (CiSHuB) (KKa).

## Conflict of Interest

The authors declare that the research was conducted in the absence of any commercial or financial relationships that could be construed as a potential conflict of interest.
